# Lessons (to be) learned from liquid biopsies: assessment of circulating cells and cell-free DNA in cancer and pregnancy-acquired microchimerism

**DOI:** 10.1007/s00281-025-01042-z

**Published:** 2025-02-01

**Authors:** Lina Bergmann, Ann-Kristin Afflerbach, Tingjie Yuan, Klaus Pantel, Daniel J. Smit

**Affiliations:** Institute of Tumor Biology, University Medical Center Hamburg-Eppendorf, Martinistraße 52, Hamburg, 20246 Germany

**Keywords:** Liquid biopsy, Circulating tumor cells, Circulating tumor DNA, Cell-free DNA, Biomarkers, Pregnancy-acquired microchimerism

## Abstract

Tumors constantly shed cancer cells that are considered the mediators of metastasis via the blood stream. Analysis of circulating cells and circulating cell-free DNA (cfDNA) in liquid biopsies, mostly taken from peripheral blood, have emerged as powerful biomarkers in oncology, as they enable the detection of genomic aberrations. Similarly, liquid biopsies taken from pregnant women serve as prenatal screening test for an abnormal number of chromosomes in the fetus, e.g., via the analysis of microchimeric fetal cells and cfDNA circulating in maternal blood. Liquid biopsies are minimally invasive and, consequently, associated with reduced risks for the patients. However, different challenges arise in oncology and pregnancy-acquired liquid biopsies with regard to the analyte concentration and biological (background) noise among other factors. In this review, we highlight the unique biological properties of circulating tumor cells (CTC), summarize the various techniques that have been developed for the enrichment, detection and analysis of CTCs as well as for analysis of genetic and epigenetic aberrations in cfDNA and highlight the range of possible clinical applications. Lastly, the potential, but also the challenges of liquid biopsies in oncology as well as their translational value for the analysis of pregnancy-acquired microchimerism are discussed.

## Introduction

During tumorigenesis somatic cells acquire severe genomic aberrations including mutations, translocations or copy number variations that drive the deregulated cell proliferation. Metastatic spread of the primary tumor is one of the major reasons for cancer mortality. The metastatic cascade includes the invasion of the tumor cell into the surrounding tissue, intravasation into the blood stream, spread to distant organs, extravasation and the colonization of a new niche. Circulating tumor cells (CTCs) are considered as blood-borne mediators of metastasis [1]. On their journey from the primary tumor to the distant site of metastasis, CTCs must adapt to the harsh conditions in the circulation and to the foreign environment at the site of extravasation. Next to their fundamental role in the biology of metastasis, CTCs can serve as blood-based biomarkers and important sources of information, as they share most genomic aberrations with the tumor. Liquid biopsy includes the analysis of CTCs and other tumor-associated products including cell-free DNA (cfDNA) from body fluids [2]. The advantages of liquid biopsies over tissue biopsies comprise the minimally invasive character of sample collection, the possibility of repeated (longitudinal) sampling and the ability to better capture the tumor heterogeneity [3]. Therefore, liquid biopsy has the potential to be employed for versatile purposes along the patient’s journey including early diagnosis, identification of targets for personalized treatment, response biomarker and detection of minimal residual disease.

The fact that CTCs migrate with an aberrant genome through the host organism and their role as biomarkers to inform about the tumor leads us to suggest that parallels may exist to fetal microchimerism (FMC) represented by fetal cells with a partial “foreign” paternal composition that migrate through the maternal organism and similarly serve as an analyte for non-invasive prenatal testing (NIPT) of the fetal genome [4]. FMC are typically stem cells, immune cells, trophoblasts, fetal nucleated red blood cells (fNRBCs), or their products including circulating cell-free fetal DNA (cffDNA) [5–9]. Fetal and maternal cells exhibit bidirectional transfer during gestation. The transfer is, however, asymmetrical, favoring the movement of fetal cells to the maternal system [10–12]. Fetal cells can pass the placenta and settle in maternal tissues during pregnancy, with their prevalence rising with increased gestational age. These fetal cells can remain in the maternal system for several years post-delivery [13–16]. FMC is detectable in various maternal tissues, including the bone marrow and organs such as the kidney, as well as blood [17–19].

Similarly to CTCs, parallels between cfDNA in cancer patients as well as pregnant women have already been reported several years ago [20]. A better understanding of the underlaying mechanisms (e.g. immuno-tolerance) in the feto-maternal field and mediation of immune-escape in cancer could be beneficial for both research areas.

In this review, we aim to summarize CTC and cfDNA biology and highlight techniques and methods developed for assessment of these analytes by liquid biopsies. Finally, we will discuss if and how advances in the oncological liquid biopsy field can be transferred to feto-maternal medicine/NIPT and vice versa.

## Biology and origin of CTCs and ctDNA

Circulating tumor cells originate from the primary tumor or distant metastasis and leave the tumor tissue either by passive shedding or active cell migration [21]. Several studies could show that CTCs harbor common genomic aberrations with the tumor tissue, however private aberrations in CTCs have also been described in the past [22]. After the CTCs leave their tissue or origin, the cells must survive in a hostile environment with absent cell-cell-connections. The shear stress present within the blood vessels and the numerous immune cells pose severe threats to the CTCs and this results in a short CTC half-life time of 1–3 h [23]. However, some CTCs adapt to these conditions, resist anoikis and seed metastasis in distant organs [24]. Several mechanisms have been identified that allow CTCs to survive, including the formation of cell clusters with platelets and immune cells, acquisition of stemness features and epithelial-to-mesenchymal transition (EMT) [25].

In 2002, Jean Paul Thiery introduced a model for metastasis involving reversible EMT, where cancer cells undergo EMT to invade and mesenchymal-epithelial transition to form epithelial metastases upon reaching distant sites [26]. Studies have shown that CTCs can exhibit both epithelial and mesenchymal markers, indicating a potential link between CTCs and EMT status [27]. The presence of EMT markers in CTCs is associated with advanced stages of cancer and poorer prognosis, highlighting their clinical significance in various cancer types, including breast, lung and pancreatic cancer among many others [28–30]. The detection and characterization of CTCs, especially those with mesenchymal features, have been highlighted as valuable for understanding cancer progression, metastasis, and treatment response [31].

In breast cancer, CTCs expressing EMT markers such as vimentin and twist are more frequently found in metastatic patients compared to those with early-stage disease. This suggests that EMT is involved in the metastatic potential of CTCs [32]. Hybrid CTCs co-expressing epithelial (keratin) and mesenchymal (vimentin) markers have been identified in patients with metastatic non-small cell lung cancer [33]. In colon cancer, CTCs with a hybrid epithelial/mesenchymal phenotype serve as effective biomarkers for guiding therapy [34]. In hepatocellular carcinoma, EMT-CTCs and circulating cancer stem cells are associated with aggressive clinicopathological features and increased risk of early recurrence, emphasizing their prognostic value in hepatocellular carcinoma management [31]. CTCs undergoing EMT, particularly those with a hybrid epithelial/mesenchymal phenotype, play a significant role in cancer metastasis. These cells are more resistant to apoptosis and have higher tumor-initiating potential, making them critical targets for monitoring and guiding cancer therapy.

Furthermore, EMT is involved in tumor immune escape by promoting immunosuppression and resistance to immune effector mechanisms [35]. CTCs exhibit a complex interplay with the immune system, particularly through mechanisms like EMT that contribute to immune escape and enhance their survival in circulation and metastatic potential [36–38]. EMT in CTCs can lead to the upregulation of immune checkpoint regulators like programmed cell death ligand 1 (PD-L1), enabling them to evade immune surveillance and promote the survival in the circulation [36]. The co-expression of PD-L1 and EMT markers in CTCs from NSCLC patients has been suggested as ‘molecular shield’ mechanism supporting immune evasion, potentially mediating resistance to immunotherapy [39]. Studies suggest that CTCs with EMT features, influenced by factors including platelet interaction and TGF-β signaling, could resist immune detection and attack, facilitating their metastatic spread and survival in the bloodstream [40]. The acquisition of stem-cell-like features by CTCs through EMT further enhances their metastatic potential and resistance to immune effector mechanism, highlighting the intricate relationship between EMT, immune escape, and CTC behavior [37, 41].

Apart from intact CTCs, fragmented tumor DNA is present in the blood stream and other body fluids of cancer patients. This cell-free tumor DNA (ctDNA) is derived from apoptotic and necrotic tumor cells or actively secreted via extracellular vesicles. Similar to the CTCs, ctDNA is diluted in healthy cfDNA mainly derived from hematopoietic cells, requiring ultra-sensitive method for detection of the minute amount [42, 43]. cfDNA is characterized by a typical bi-modal fragmentation pattern that corresponds to the length of DNA wrapped around a histone and is a consequence of DNA degradation during apoptosis. ctDNA generally shows a smaller fragment length distribution compared to cfDNA, while longer fragments are derived from necrotic cells [44]. The half-life time of ctDNA ranges between 1 and 2 h and, thus, ctDNA enables real-time monitoring of the disease [45, 46].

To conclude, CTCs resemble a cell population with a fundamental role in the biology of cancer metastasis and unique properties in regard to migration and immune escape. Both CTCs and ctDNA are continuously shed from the tumor and represent its genomic aberrations. Thus, the use of liquid biopsies and specifically CTCs and ctDNA analysis provides further insight into clonal evolution of the tumor. Liquid biopsy analytes can provide a more heterogeneous picture of the tumor than a single tissue biopsy can, as they are more arbitrarily shed from throughout the tumor which might be missed by a single biopsy. This has for example been shown by longitudinal analysis of CTCs in melanoma and shows intra-patient heterogeneity and its somatic evolution [47].

## Analysis of circulating tumor cells for cancer research and diagnostic

**Fig. 1:**
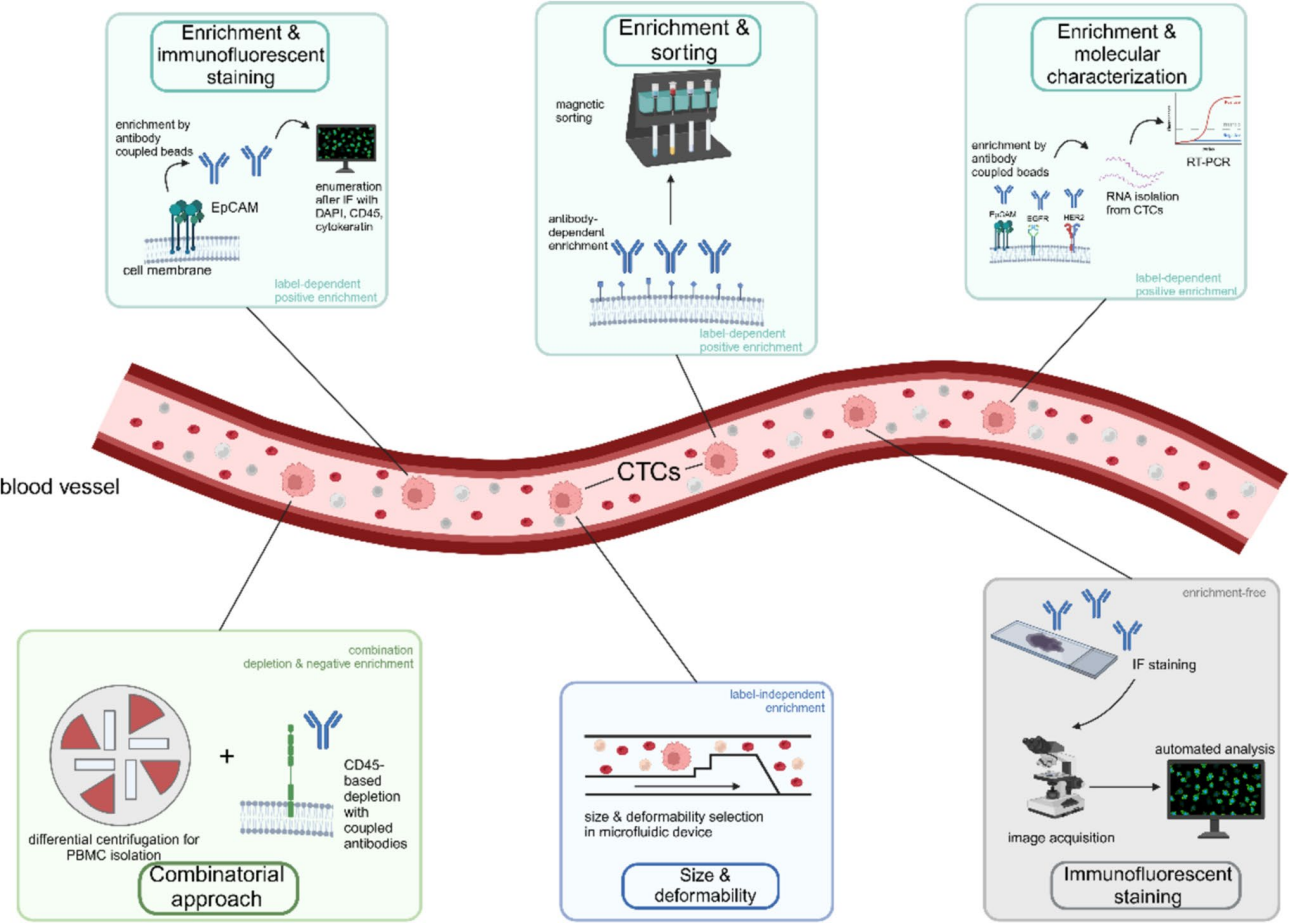
Schematic overview of CTC capture methods with label-dependent enrichment (enrichment & immunofluorescent staining, enrichment & sorting, enrichment & molecular characterization), the combination of negative enrichment and differential centrifugation (combinatorial approach), label-independent enrichment (size & deformability) and enrichment-free methods (immunofluorescent staining). Label-dependent methods are highlighted in green, the label-independent method is highlighted in blue and the enrichment-free method is highlighted in gray. CD45 – cluster of differentiation 45; CTCs – circulating tumor cells; EGFR – epidermal growth factor receptor; EpCAM – epithelial cell adhesion molecule; HER2 – human epidermal growth factor receptor 2; MACS – magnetic-activated cell sorting. The Figure was created using BioRender.com

In peripheral blood, the rare CTCs are among millions of leukocytes and billions of red blood cells [48]. Therefore, an enrichment of the CTCs is required for subsequent molecular analysis of tumor-specific features. Over the past decades, a plethora of methods has been developed for CTC enrichment from body fluids, predominantly blood. These methods can be divided into positive and negative enrichment, depending on whether tumor cell specific markers are used for selection or leukocyte markers are used for depletion. Among the positive enrichment methods, label-dependent methods rely on the expression of specific cell surface antigens, while label-independent methods mainly rely on physical properties such as tumor cell density, size, deformability and electric charge. A schematic overview of the most commonly used enrichment methods is given in Fig. 1. After enrichment, the CTC concentration can be increased by several orders of magnitude to a few tumor cells in hundreds to thousands of leukocytes [49]. CTCs can then be detected and analyzed for their molecular properties.

Pre-analytical factors to consider for liquid biopsies including blood preservation, storage or transport, as well as processing are reviewed elsewhere [50–53]. However, due to the influence of pre-analytical factors it is important to carefully plan clinical studies or diagnostic routines with consideration of these factors.

### Positive enrichment

#### Label-dependent enrichment

Label-dependent enrichment methods are mainly immune-affinity based and require the identification of cancer-specific cell surface antigens. While no universal marker is available that is able to capture the plethora of CTC subtypes across different cancer entities, the epithelial cell adhesion molecule (EpCAM) has emerged as the most frequently used target in solid (epithelial) tumors [54]. The CellSearch system (Menarini) is the first FDA-approved system for CTC enrichment and detection. EpCAM expressing cells are enriched by antibody-coupled magnetic beads in an automated workflow and are detected and enumerated after immunocytochemistry staining. The CellSearch system is approved for multiple tumor entities including breast, prostate and colorectal cancer [55]. The recovery rates range between 76 and 82% and heavily depend on the level of EpCAM expression in the CTCs [56, 57]. EpCAM-based enrichment methods, including CellSearch, often miss EpCAM-low and EpCAM-negative CTCs, leading to an underestimation of the total CTC count [58–60]. In contrast to CellSearch, the AdnaTest (Qiagen) utilizes additional targets for CTC enrichment in addition to EpCAM, with the aim to enrich a more heterogeneous fraction of CTCs. The cell separation can be performed manually or automated and the recovery rates of up to 100% can be achieved [61, 62]. The comparison of AdnaTest and CellSearch confirmed that the addition of antigens for CTC enrichment provides complementary prognostic information [63]. Magnetic-activated cell sorting (MACS, Miltenyi Biotec) is based on the principle of immune-magnetic enrichment [64] similar to AdnaTest and CellSearch but offers more flexibility with regard to the antigen for enrichment. For example, Bartkowiak et al. identified CDCP1 as a cell surface marker of breast cancer cells with mesenchymal features and were able to enrich also EpCAM-negative CTCs from patient samples [65]. The recovery ranges of magnetic-activated cell sorting highly depend on the specific antigen chosen. While the most common label-dependent applications are based on antibodies, other selective capture probes are being developed including peptides or aptameres [66, 67]. Novel methods also harness tumor specific membrane glycoproteins for less biased enrichment [68].

#### Label-independent enrichment

Label-independent enrichment methods do not require a specific marker but rather rely on physical properties of tumor cells such as size, density and deformability. The use of intrinsic physical properties of the tumor cells does not require any dye or antibody and thus enables a less biased capture of CTCs. Usually tumor cells are larger, denser and more rigid than leukocytes, allowing these systems to also isolate EpCAM-negative CTCs as well as CTCs with a more mesenchymal phenotype [69]. However, exceptions are found in certain tumor entities when smaller CTCs are present as observed in patients with small cell lung cancer and prostate cancer [70]. Cell membrane stress, sample loss and clogging are common disadvantages of enrichment methods that are based on the physical properties of the cells [71].

An FDA-cleared system for size- and deformability-based enrichment is the Parsortix device (Angle Inc.). CTCs are enriched in a microfluidic cassette with a narrow gap that allows red blood cells and leukocytes to pass but traps larger tumor cells. Although fixation leads to more rigid cells and hence, improved capture, fixation is not mandatory and viable CTCs can be enriched for functional analysis. The recovery rate ranges between 69 and 90% [69]. Additional filtration methods with a pore size of 5–7 μm that rely on cell size and deformability are the CROSS chip with a recovery rate of 70%, the CellSieve with a recovery rate of 60–80% or the ISET system with a recovery rate of up to 95%, among others [72–74]. Microfluidic spirals harness inertial and centrifugal forces to separate potential tumor cells from leukocytes. Studies have demonstrated that these spirals can achieve recovery rates of more than 85% and allow the isolation of heterogeneous CTC populations including stem-like CTCs [75, 76]. Additional methods also employ ultrasound (acoustophoresis) or electric fields (dielectrophoresis) for CTC enrichment [77, 78].

### Negative enrichment / depletion

Negative enrichment (also referred to as depletion) strategies are based on the depletion of host cells (particularly immune cells) and have the advantage of not introducing any bias in the enrichment of CTC subpopulations that occurs when selecting tumor-specific features for enrichment. The RosetteSep Kit (STEMCELL Technologies) utilizes immune cell surface markers such as CD45 for depletion by immunodensity-based separation. Selected cells are crosslinked to red blood cells and removed by density gradient centrifugation, resulting in unmodified enriched CTCs that can be used for cultivation or molecular analysis [79].

The CTCeptor is based on a similar strategy but combines isolation of peripheral blood mononuclear cells and CD45-based depletion in an automated workflow that enables parallel purification of enriched CTCs and plasma. The CTC fraction showed considerable heterogeneity regarding cell size and EpCAM expression, indicating the advantages of negative enrichment. Recovery rates of 82–93% were reported from spike-in experiments with different cancer cell lines [80]. Similarly, the microfluidic CTC-iChip removes leukocytes using an antibody-cocktail against markers such as CD45, CD3 and CD16 among others; however, cell separation is performed with the magnetic sorter chip and not by centrifugation. With this method a recovery rate of 89% of spiked-in CTCs was achieved, while more than 99.9% of leukocytes, red blood cells and platelets were successfully removed [81].

### Enrichment-free methods

As positive enrichment might miss relevant CTC subpopulations and negative enrichment methods bear the risk to lose CTCs due to unspecific binding, platforms have been developed that operate without CTC enrichment but directly proceed with CTC detection. The Epic Platform (Epic Sciences) is based on the immunofluorescence-based analysis of all nucleated cells from a blood sample and comprises automated CTC detection using cell morphology and marker expression. A mean recovery rate of 88% was reported with this device [71]. In a similar manner, the automated RareCyte platform detects CTCs by imaging immunofluorescence-stained peripheral blood mononuclear cells that have been isolated before by density gradient centrifugation. This system also allows the picking of single cells for in depth molecular analysis and recovery rates are about 80–90% [82].

Wang et al. recently reported a method of CTC detection after partitioning instead of enrichment. Similar to digital PCR, peripheral blood mononuclear cells were dispersed into multiple droplets and tumor-specific targets were detected by PCR in the individual droplets. Using this method, the researchers reported recovery rates between 75 and 94% in spike-in controls and were able to identify non-small cell lung cancer patients with treatment-relevant epidermal growth factor receptor mutation [83].

### Methods for CTC detection

Even after enrichment, cell fractions will still contain hundreds to thousands of leukocytes or other mononuclear cells next to the CTCs [3]. Therefore, an additional step is required to detect single CTCs. The most commonly applied method for CTC detection is immunocytochemistry staining. Typically, this includes CD45 as a negative marker and cytokeratin as a positive marker for (epithelial) tumor cells, but other markers might be used depending on the tumor entity. Next to fluorescence signals, features of cell morphology such as cell dimensions, nuclear to cytoplasmic ratio, or nuclear shape can be analyzed to identify CTCs as included in the workflow of the Epic CTC platform [71]. Microscopy images are often examined manually after automated pre-selection which causes inter-operator variability in data analysis. Recent advances in AI-assisted image analysis are integrated into the CTC detection workflow to increase standardization and quality of the data. Modern convolutional neural networks are able to differentiate between CTCs and non-CTCs with an area under the curve of 0.99 [84].

The use of PCR-based assays is another method to detect CTCs and is integrated into the AdnaTest workflow, for instance. Assays are either designed for tissue or tumor-specific transcripts and mutations [85]. In contrast, the EPISPOT assay is specifically designed for the detection of viable cells in short-term culture based on the secretion of specific proteins from potential CTCs. Enriched CTCs are cultivated for 48 h in antibody-coated plates. The protein of interest is chosen based on the tumor entity, for instance prostate-specific antigen in prostate cancer. After cell removal the captured marker proteins are fluorescently labeled and immunospots are counted [86].

### Methods for molecular analysis of CTCs

Following CTC detection, an advanced analysis and characterization of molecular features of the CTCs offers the possibility to identify targets for personalized treatment, monitor resistance mechanisms or gain fundamental insights into CTC biology. Fluorescence in situ hybridization is a standard method to assess copy number variations in tumor cells. Similarly, fluorescence in situ hybridization can be used to study copy number variations or rearrangements in CTCs to identify known aberrations that are associated with prognosis of response to therapy such as *TMPRSS2:ERG* fusion in prostate cancer or *ALK* translocations in non-small cell lung cancer [87, 88]. However, this method does not allow the identification of novel genomic aberrations. For this purpose, CTCs can be subjected to next generation sequencing applications. Due to the low cell numbers, whole genome amplification usually proceeds the library preparation process. Shallow whole genome sequencing of DNA acquired from CTC whole genome amplification is sufficient to identify copy number variations, for instance actionable targets such as *ERBB2* and *BRCA2* in breast cancer [89]. Due to potential replication errors introduced during whole genome amplification, the calling of single base mutations from single cell CTC sequencing data is challenging, while digital PCR offers the possibility to study known mutations in small sample quantities with high sensitivity [90, 91]. DNA methylation analysis of bulk or single CTCs using bisulfite-sequencing techniques can reveal additional epigenetic information. Single cell methylome analysis in CTCs identified, for example, early immunosuppressive methylation patterns that facilitate prostate tumorigenesis [92].

Reverse transcriptase-PCR is the simplest method of transcript analysis in CTCs. Using multiplex digital PCR assays, also the absolute quantification of transcripts in enriched CTC fractions is feasible [93]. Single cell resolution can be achieved by in situ hybridization techniques for RNA detection through imaging, such as microRNAs [94]. Multiplex single-cell in situ detection of specific transcripts is also possible by padlock probe-based approaches that harness in-cell rolling circle amplification and fluorescent probes [95]. Single-cell RNA sequencing of enriched CTCs enables the detailed analysis of CTC heterogeneity and offers the chance to identify novel drivers of metastatic spread. In pancreatic cancer, single-cell analysis in CTCs identified stemness genes and onco-fetal proteins as contributors to metastatic spread, which could serve as therapeutic targets to decrease the metastatic capacity of CTCs [96].

Apart from multicolor immunofluorescence staining, proteome analysis in enriched CTC is still challenging. Multiplex analysis of up to 40 proteins is possible in single CTCs with techniques such as surface-enhanced Raman spectroscopy, mass cytometry or microfluidic western blot [97–99]. All methods require prior knowledge about the proteins of interest, but recent advances in the field of single-cell proteomics give hope that single cell proteome data will be acquired from CTCs in the near future [100].

After enrichment of viable cells, CTCs can also be cultivated in vitro or used to establish xenotransplantation models in vivo [101]. Several CTC lines representing different tumor entities have been generated from patient samples so far and serve as models to study CTC biology or drug susceptibility [102]. However, the establishment of long-term stable CTC lines is rather difficult and only a few stable CTC lines exist to date [102].

### Clinical applications of CTC analysis

The detection and analysis of CTCs is not only relevant to understand the biology of tumor cell dissemination and metastasis but also to identify novel biomarkers for clinical applications.

The use of CTCs for screening and early diagnosis of cancer is generally difficult due to the low CTC numbers observed at early disease stages. While the detection of CTCs during lung cancer screening in a high-risk population was highly specific for subsequent lung cancer diagnosis, the sensitivity of the analysis was poor and did not allow an accurate prediction of lung cancer patients [103]. Similarly, another prospective study in healthy individuals and colorectal cancer patients before colonoscopy indicated that the detection of CTC robustly predicted the presence of colorectal cancer [104]. Efforts to increase the sensitivity for early diagnosis includes the analysis of larger blood volumes or the combination of multiple biomarkers.

Once cancer has been diagnosed, CTCs can be used as prognostic biomarkers in multiple cancer entities. The CTC count as determined by EpCAM-based enrichment has been shown to be associated with progression-free and overall survival in patients with breast, colorectal and pancreatic cancer among others [7, 105, 106]. Due to their prognostic value, CTCs can also serve as surrogate end points for survival in clinical trials. In metastatic castration-resistant prostate cancer, CTC enumeration was explored as surrogate marker in a phase 3 trial of abiraterone acetate and in combination with serum lactate dehydrogenase the CTC count at 12 weeks after therapy fulfilled all criteria as surrogate for overall survival [107].

Apart from the detection and count of CTCs, molecular analysis can inform about targets for personalized treatments and serve as a predictive biomarker. For instance, PD-L1 expression has been identified as a relevant marker for immune checkpoint inhibition therapy. The PD-L1 expression on CTCs is a predictive biomarker with the potential to identify responders beforehand or to monitor the response to the treatment in multiple tumor entities [108, 109]. In castration-resistant prostate cancer, the expression of androgen receptor splice variants is a known resistance mechanism to androgen receptor signaling inhibitors. The detection of androgen receptor splice variants in CTCs was significantly associated with patient outcome after androgen receptor signaling inhibitors and detection of androgen receptor splice variants at baseline was a predictive marker to guide the decision between androgen receptor signaling inhibitors and chemotherapy [110]. In breast cancer patients, the analysis of HER2 expression in CTCs can provide complementary information to tissue expression and predicts response to Lapatinib treatment [111]. In patients with cancer of unknown primary, CTC genomic analysis offers the possibility to gain insight into the tissue of origin or identify relevant targets for treatment [112].

The subclinical persistence of tumor cells in patients treated with curative intent is known as minimal residual disease (MRD). In breast cancer patients, the detection of CTCs even 5 years after diagnosis was identified as an independent prognostic factor for MRD and late disease recurrence [113].

Taken together, the choice of enrichment strategy is the first and most critical step for subsequent CTC analysis. Once CTCs have been detected successfully, they offer multiple analytes for the assessment of cancer cell genotype and phenotype markers that can be utilized for diverse clinical applications. A summary of the presented techniques is available in Table 1.

**Table 1:** Overview of selected liquid biopsy isolation approaches targeting CTCs presented in the manuscript

Method	Input Material	Description	References
Positive selection			
Immunoaffinity-based enrichment	Whole blood	Immunoaffinity-based enrichment and subsequent immunofluorescent staining	[55–57, 61–65]
Size and deformability-based	Whole blood	Enrichment based on size & deformability e.g. microfluidic devices, cell filters	[69, 72–74]
Negative selection / depletion			
Crosslinking of immune cells with red blood cells	Whole blood	Enrichment based on the depletion of immune cells by cross-linking with red blood cells (different antibody depletion cocktails available)	[79]
Depletion of CD45 + cells	Whole blood	Enrichment by gradient density centrifugation followed by depletion of CD45 + cells	[80]
Combined approaches			
Size and deformability-based positive selection combined with depletion	Leukapheresis product / Whole blood	Enrichment based on the combination of size-based inertial separation, incubation with antibodies against CD45, CD66b, CD16, CD3 for subsequent immunomagnetic negative selection through a microfluidic permeability-enhanced magnetic sorter	[81]
Enrichment-free approaches			
Enrichment-free	Whole blood	Enrichment-free approach coupled with immunofluorescent staining	[71, 82]

## Methods for cfDNA analysis and their clinical application

ctDNA analysis is currently already used for several clinical applications, for example initial diagnosis of a specific entity or monitoring of the disease in several body fluids. For this, a multitude of different methods is employed, including, but not exclusive to, droplet digital PCR, sequencing approaches and methylation analysis. Many companies provide tests to either capture specific mutations to make informed therapeutic decisions, perform whole genome sequencing or whole exome sequencing to gain insights on the tumor or to monitor the temporal heterogeneity of the disease.

ctDNA, and cfDNA in general, as well as CTC analysis is subject to several challenges. Apart from the pre-analytical factors and low abundance of analytes in blood, the quality control and standardization can be challenging. Efforts are being made to certify laboratories and adhere to a common standard for liquid biopsies, both including governmental accreditations according to ISO (EU) or CLIA (US) and standardization efforts from scientific communities worldwide [114–117].

### Droplet digital PCR

Many cancer entities harbor specific point mutations, such as BRAF V600E for melanoma, H3 K27M for diffuse midline glioma or KRAS G12 or G13 in colorectal carcinoma [118–121]. Specific single-base mutations can accurately be detected by highly sensitive and specific methods such as droplet digital PCR. Here, a single-base specific probe attaches to the target DNA and only when the DNA is elongated during the PCR reaction, the quencher of the probe detaches, and the probe can emit a fluorescent signal. In turn, the method allows for a highly specific detection and quantification of signals and can thus confirm the presence of mutated sequence in the sample [122]. Copy number variation analysis is also possible using droplet digital PCR, when using specific probes against a reference gene, however this is less commonly used in clinical applications [123]. Droplet digital PCR tests are commercially available for many hotspot mutations in cancer, with some tests even approved for in vitro diagnostics.

In the clinical routine, droplet digital PCR is for example used to detect epidermal growth factor receptor T790M mutations in cfDNA from plasma of non-small cell lung cancer patients. The presence of the mutation then allows to make the informed decision for a treatment with Osimertinib and leaves the possibility to monitor the tumor evolution in regard to the acquisition of this resistance mutation with a minimally invasive and easily feasible method [124]. Diffuse midline gliomas are often hard to perform surgery on as they are located in areas of the brain that are difficult to access and the risk for damage to crucial structures by surgical intervention or biopsy is high. Nevertheless, it is important to correctly diagnose the tumor entity in order to decide on the appropriate treatment for the patient. Droplet digital PCR can be used in this case and has been shown to work for mutation detection, especially in cerebrospinal fluid [125]. Currently, many clinical trials are employing droplet digital PCR for quantification, mutation detection and biomarker discovery [126].

### Next generation sequencing

In addition to the detection of single known hotspot mutations in cancer, more personalized (tumor-informed) approaches can be used. For example, DNA from tumor tissue can be sequenced using next generation sequencing techniques. These use sequencing by synthesis approaches, where the complementary strand of DNA is built, and each dNTP integrated is detected [127]. This can also be achieved using cfDNA derived from liquid biopsies, often in the form of sequencing panels, where only specific genes are covered that match the mutational landscape of the (primary) tumor tissue. These gene panels vary in size, investigating from 10 up to over 500 genes in a single run, often covering not only mutations, but also fusions and amplifications of specific genes relevant for cancer diagnostics [128–130]. A high sequencing depth is required to confidently call mutations that can in turn be targeted with specific therapies and potentially improve the outcome of patients. As the fraction of ctDNA and the yield in liquid biopsies is lower compared to genomic DNA from tissue, the required input for the high coverage sequencing approaches cannot always be reached [115, 131]. Low coverage whole genome sequencing or shallow sequencing is then employed to perform copy number variation analysis [132, 133]. The DNA is sequenced and aligned to the human reference genome, and then losses or gains of focal regions or chromosomal arms are inferred and plotted, which indicates the presence of tumor DNA and can be used for diagnosis in case of specific alterations or monitoring.

Next generation sequencing panels can be used, similarly to droplet digital PCR, to detect mutations that are associated with treatment resistance, however on a larger scale compared to targeted methods including droplet digital PCR. In non-small cell lung cancer, a sequencing panel of 77 genes has been used to reveal tyrosine kinase inhibitor resistance to adequately identify candidates for tyrosine kinase inhibitor treatment [134]. This was also shown in other cancer entities like colorectal cancer, in which expected frequent mutations like KRAS, but also other, potentially actionable, mutations were found [135]. Moreover, for patients with tumor-predisposition syndromes like Li-Fraumeni, cfDNA sequencing approaches can be used to detect cancer earlier than common medical imaging methods and thus help treat the patient as early as possible and improve their outcome [136].

Low coverage whole genome sequencing data can also be used to perform fragmentation analysis and nucleosome profiling, which allows the distinction between nucleosome-protected regions and -unprotected regions that in turn can be used for cancer detection and give insights into the cell-of-origin. This can for example be used for subtyping of the estrogen receptor in breast cancer patients, monitoring and to ultimately make informed therapeutical choices [137].

Several hundreds of clinical trials are currently underway worldwide, using ctDNA in cancer research. The cancer entities vary as well as the study goal – ctDNA is used for monitoring, initial diagnosis or minimal residual disease detection [138].

### Methylation analysis

DNA methylation analysis is used to distinguish cancer entities or subtypes from each other. Methylation is routinely analyzed for tissue in many pathology departments using either Illumina microarrays or reduced representation bisulfite sequencing. The methylation arrays are well established and nowadays and cover over 900k CpG sites, however they require a large amount of DNA and are thus not a method of choice for cfDNA from liquid biopsies yet [139]. Bisulfite sequencing, on the other hand, is applicable and used for the analysis of cfDNA [140]. By treating cfDNA with bisulfite, unmethylated cytosines are converted into uracil by deamination, which are later turned into thymidine during the sequencing process and can then be read out and analyzed.

Analysis of differentially methylated regions can be used to distinguish between malignant and benign diseases in the same organ, for example pancreato-biliary cancers and pancreatitis by enriching for the most variable CpG sites [141]. Methylation analysis of cfDNA has also been demonstrated to successfully detect the primary cancer in patients with cancer of unknown primary [142].

Not only methylation of DNA, but also histone modifications in the form of, for example, histone 3 lysine 4 trimethylation (H3K4me3) can be used for detection of cancer [143]. Both the methylation of histones and of DNA are intricately linked, influencing cancer development and progression [144], representing novel targets for diagnostic tools.

### Multimodal analysis of epigenetic and genetic features

As a combination of both sequencing and methylation analysis from the same sample, third generation sequencing like Nanopore sequencing can be employed. Both markers can be acquired simultaneously as the DNA is analyzed in its native state. The DNA is drawn through a nanopore embedded in a membrane and each passing base specifically alters the electric current through the pore, which can then be read and translated into the sequence with its methylation [145]. Nanopore sequencing was initially developed for long-read sequencing but has since been adapted for many approaches, including short cfDNA analysis, and is developed into diagnostic tools [146].

For example, in a study by Afflerbach et al. it has been shown that Oxford Nanopore Sequencing technology can detect and analyze ctDNA in cerebrospinal fluid of patients with central nervous system tumors [147]. In this study, the copy number variation profile was analyzed for the presence of aberrations, indicating the presence of tumor-derived DNA, while the methylation data were analyzed for accurate diagnosis of the entity, highlighting the use of both sequence and methylation for integrated diagnosis.

In summary, modern PCR and sequencing techniques enable in depth analysis of cfDNA that is comparable to the genomic analysis of tumor tissue. Being utilized as biomarkers in multiple ongoing clinical trials, cfDNA is on its way to routine clinical application for diagnosis and monitoring of cancer patients. A summary of methods presented in this chapter is available in Table 2.

**Table 2:** Overview of selected liquid biopsy approaches targeting cfDNA presented in the manuscript

Method	Input Material	Description	References
Digital droplet PCR	cfDNA from plasma	Analysis of mutations, structural variants and CNVs	[122–126]
Next Generation Sequencing	cfDNA from plasma	Analysis of mutations, structural variants, CNVs, fragmentation	[127–130]
Methylation analysis following conversion	cfDNA from plasma	Analysis of methylation by bisulfite sequencing or on targeted microarrays (> 900k CpG sites)	[139–142]
Nanopore Sequencing	cfDNA from plasma	Analysis of CNVs & native methylation analysis	[145–147]

## Fetal microchimerism – influence on maternal health and their use for diagnostics

FMC has been associated with both, positive and negative effects on maternal health. It may facilitate tissue repair and enhance regulatory T cell proliferation to regulate immune responses and avert fetal rejection [11, 148]. Moreover, FMC is associated with lower breast cancer risk and offers protection against various malignancies [149–152]. However, increased FMC is noted in autoimmune disorders including systemic lupus erythematosus and rheumatoid arthritis [11]. The augmentation of FMC has been reported in preeclampsia [12].

FMC possesses unique genetic and epigenetic characteristics, making it highly valuable for NIPT. These distinctive features provide understanding of fetal and maternal well-being, similar to the detection of circulating tumor cells (CTCs), without the need for invasive procedures. Sequencing methodologies facilitate the detection of fetal aneuploidies and various genetic disorders by distinguishing fetal DNA from the maternal DNA component [153, 154]. Coordinated allele-aware targeted enrichment sequencing (Coate-seq) enhances the accuracy of detecting fetal-specific genetic variations, including aneuploidies and microdeletions, by analyzing cfDNA fragment lengths and meiotic recombination patterns [154].

Additionally, fetal DNA features different methylation profiles in comparison to maternal DNA, especially those observed at CpG islands, which can serve as fetal-specific epigenetic signatures for identification. For example, the characteristic hypomethylation of fetal DNA compared to maternal DNA is a distinguishing feature that can be applied for NIPT [155, 156]. Specific differentially methylated regions (DMRs) have been identified as potential biomarkers for detecting fetal aneuploidies. Techniques such as methylated DNA immunoprecipitation (MedIP) combined with next-generation sequencing (NGS) have been employed to analyze these regions, providing a high accuracy in detecting conditions like trisomy 21 [157, 158]. The presence of 5-hydroxymethylcytosine (5hmC) in fetal DNA, especially in chorionic villi, represents another epigenetic marker that can be utilized to enrich fetal DNA in maternal plasma. This approach reduces the required number of sequencing reads, making the process more cost-effective [155].

fNRBCs provide a complete genetic profile, making them highly reliable for genetic analysis in NIPT [159, 160]. Advanced microchip technologies have been developed to effectively isolate fNRBCs from maternal blood, enhancing the accuracy and efficiency of NIPT [159]. Novel developments using aptamer-based interfaces (HUNTER-Chip) or antibody-labelled microfluidic chips (FETAL-Chip) significantly improve the capture and release efficiency of circulating fetal cells, facilitating detailed genetic analysis [160, 161].

Fetal cells can be characterized by specific cell surface markers that differ from those of maternal cells. High-dimensional cytometry (CytoF) and immunofluorescence microscopy are utilized to identify these markers, which may include fetal immune or stem cell-specific markers [162]. As FMC are typically stem or immune cells that exhibit a mesenchymal phenotype and express pluripotent stem cell associated markers (e.g. CD34, OCT-4, Nanog, Rex-1) [163, 164]., their unique phenotype can be used to distinguish them from maternal cells [165].

The unique genetic and epigenetic characteristics of fetal microchimeric cells provide a promising foundation for non-invasive prenatal diagnosis, offering a safer alternative to traditional invasive methods. However, several challenges remain to be addressed. For example, the low concentration of cffDNA in the context of high maternal DNA background leads to a more challenging detection process, requiring advanced sequencing and bioinformatics technologies to accurately identify fetal-specific markers [156, 166]. Furthermore, the variability in methylation patterns among individuals and tissues can affect the reliability of epigenetic markers, warranting careful validation and standardization of testing methods [157, 167]. The complexity to distinguish fetal DNA from maternal DNA and the variability of epigenetic markers present ongoing challenges that require further research and technological advancements.

In diagnostic studies, ddPCR provides a high sensitivity and specificity for detecting low-abundance DNA, making it suitable for identifying FMC in maternal blood and tissue. ddPCR allows for more precise detection of microchimerism and simple, yet sensitive diagnostic of single-gene point mutations in cffDNA within maternal blood, offering a less invasive alternative compared to traditional methods such as amniocentesis and chorionic villus sampling thereby reducing the risk of iatrogenic fetal loss [168–172]. A 100% sensitivity and specificity in detecting specific gene mutations, such as those associated with achondroplasia has been reported [173]. Additionally, nested PCR has been utilized to amplify fetal DNA from maternal samples, facilitating the detection of fetal sex and genetic anomalies [174]. Quantitative PCR (qPCR) is a commonly used technique for detecting FMC presence through fetal-specific gene polymorphism analysis. This method exhibits significant sensitivity and specificity in identifying FMC within maternal peripheral blood mononuclear cells (PBMC) [13].

Fluorescence in situ hybridization (FISH) enables the identification of male nuclei in female tissue sections using Y chromosome-specific probes. This technique has been effectively utilized in fetal cell microchimerism studies in different paraffin-embedded tissues, including the skin, lung, thyroid, adrenal glands, lymph nodes, among others [175–178]. Fetal circulating trophoblasts can be isolated from maternal peripheral blood samples using microfluidic positive selection techniques (e.g. anti-EpCAM) of maternal blood [179]. Flow Cytometry and MACS can enrich fetal cells from maternal peripheral blood, allowing identification of specific cell types, including as trophoblasts and fetal erythroblasts [174]. In summary, there is a great variety of methods employed for NIPT that each present valuable information yet come with challenges.

## Synergies between liquid biopsies in cancer research and prenatal testing

**Fig. 2:**
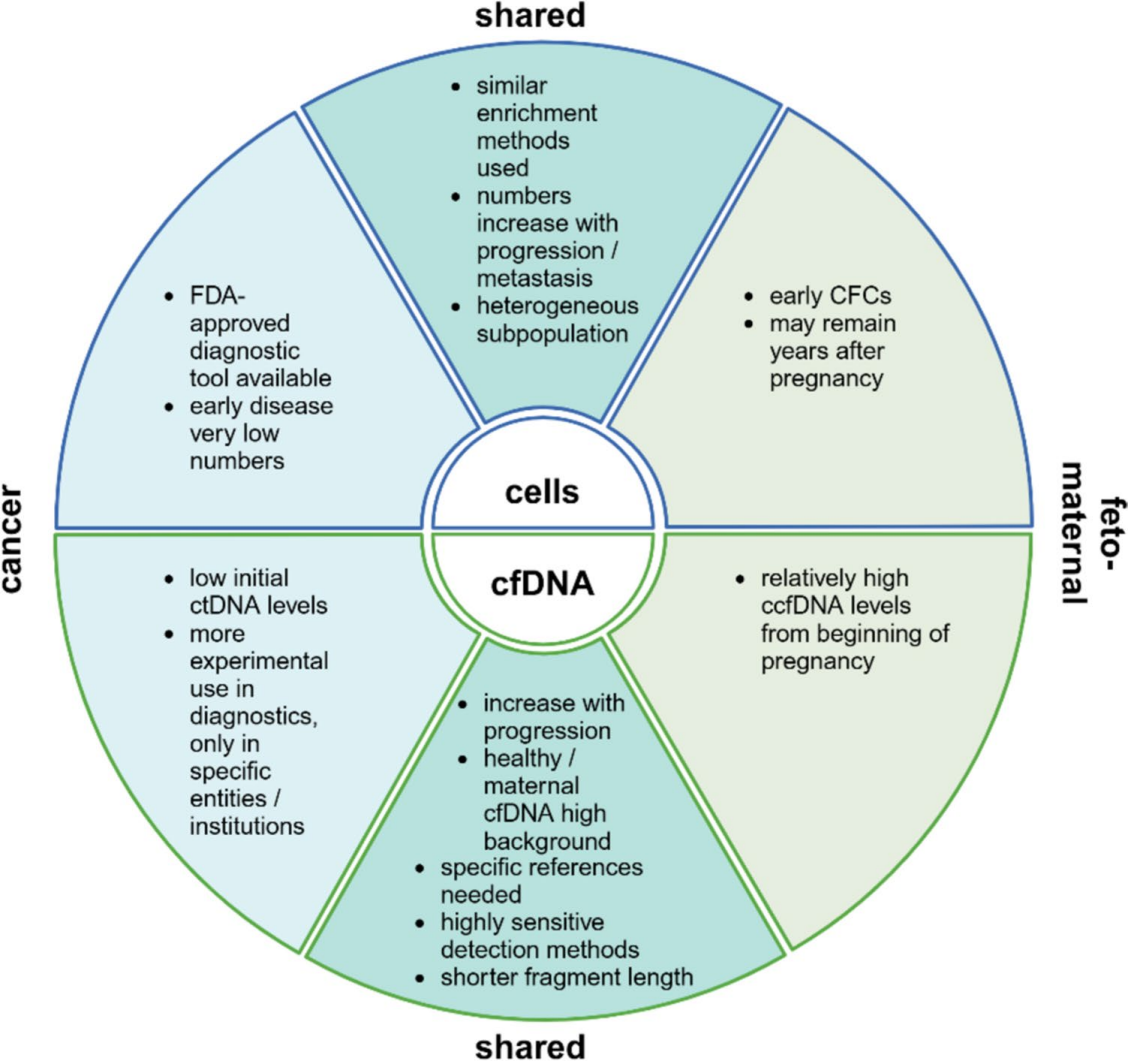
Diagram depicting differences and similarities between cell-based (surrounded by blue border) and cell-free (surrounded by green border) approaches in cancer and feto-maternal medicine. Cancer-specific statements are displayed in light blue, shared statements in cyan and feto-maternal-specific statements in light green. ccfDNA – circulating cell-free fetal cell; CFC – circulating fetal cells; cfDNA – circulating cell-free DNA; ctDNA – circulating tumor DNA; FDA – US Food and Drug Administration. The Figure was created using BioRender.com

In general, the use of non-invasive DNA and cell-based assays represents a valuable tool for clinical testing in oncology as well as in feto-maternal medicine with regards to NIPT **(**Fig. 2**)**. In cancer patients, the minimally invasive detection of genetic aberrations facilitates diagnosis, monitoring of disease and treatment response, and personalized treatment choice. In prenatal medicine, minimally invasive analysis of cffDNA and circulating fetal cells reduces the number of invasive diagnostic tests, which are associated with higher risks for mother and fetus [180]. In addition, it enables the diagnosis of rare monogenic diseases that can be treated *in utero* [181]. However, it is important to use the advancement of methods in a responsible manner and to consider ethical implications when performing NIPT (discussed in [182–184]).

As outlined before, genetic analysis of CTCs and ctDNA is used to identify point mutations, copy number aberrations, translocations/fusions and methylation patterns. In the context of NIPT, the relevant genetic aberrations include aneuploidies, microdeletions/-duplications and point mutations. While the detection of trisomies and sex chromosome aneuploidies is commonly performed on cffDNA, the detection of smaller copy number variations is currently not recommended due to the low positive predictive value [185]. Both applications require reliable detection of single nucleotide mutations for the identification of monogenic congenital disease or treatment-relevant targets but also the detection of large structural aberrations, such as translocations in cancer patients or trisomies in fetal genomes.

Additionally, both applications require highly sensitive analysis methods as the frequency of circulating “foreign” cells and the concentration cell-free DNA are low, especially at the beginning of tumor growth or in early pregnancy. In cancer, ctDNA levels in most cases correlate with tumor size, meaning that the concentration increases when the disease progresses [186]. While the ctDNA concentration in blood at early stages ranges from 0.01% up to 1%, ctDNA concentrations in patients with advanced cancers are significantly higher and may exceed 10% or even 40% [3]. Similarly, CTCs are detected at low frequencies in early disease and the CTC count rarely exceeds 1–3 cells/ml blood [104]. During disease progression and when tumors metastasize CTC counts may rise to 10->100 cells/ml blood [22, 104].

Circulating fetal cell levels have been reported to range between 2 and 6 fetal cells/ml of maternal blood in the second trimester and can be found as early as 4–6 weeks of gestation [187]. In maternal circulation, cffDNA fractions of about 6–11% can be detected at the end of the first trimester and increase further with gestational age [188, 189]. Both, ctDNA and cffDNA, are characterized by a shorter fragment length compared to the healthy cfDNA background [190]. This can be used in both fields of application to enrich relevant fragments and detect variants with increased sensitivity [185]. Among all applications in cancer research, detection of minimal residual disease requires the most sensitive methods to detect tumor-derived mutations when no disease is clinically visible. cfDNA is the analyte of choice in this setting as the available methods allow for sensitive detection of down to 0.01% ctDNA [3]. Therefore, many strategies to increase the sensitivity of ctDNA testing have been developed in recent years and these may also enhance the accuracy of NIPT.

While mutations in CTCs and ctDNA can be detected by whole genome or exome sequencing, more sensitive mutation calling is possible with the use of targeted sequencing panels or PCR assays. To design such assays, genetic characterization of the tumor tissue is required to select suitable mutations [3]. Similarly, the detection of cffDNA may require the analysis of parental DNA for accurate variant calling. Especially the detection of maternally inherited mutations remains difficult and requires more complex bioinformatics for data analysis [191]. However, in the case of a male fetus the detection of Y-chromosome sequences can be used to identify cffDNA by a single marker that is currently not possible to the same extent in most applications in cancer research [192].

In cancer as well as for NIPT, the reduction of false-positive and false-negative test results is an important goal. In NIPT, false-positive results are more common than false-negatives, the reasons for this include maternal copy number variation or cancer and placental mosaicism [193, 194]. For the detection of mutations in cancer, the presence of mutations in non-malignant hematopoietic cells known as clonal hematopoiesis of intermediate potential can cause false-positive results. This limitation can be overcome by parallel analysis of (genomic) leukocyte DNA from buffy coat [195]. For both applications, it is essential to distinguish between cases with a true-negative test result and cases with too little amount of ctDNA or cffDNA for variant calling. Therefore, the estimation of the fraction of ctDNA or cffDNA in the total cfDNA is a useful tool to evaluate negative results. On the technical level, standardized pre-analytical workflows are required to establish evidence-based guidelines and ensure robust performance of the biomarkers [196]. The routine clinical application of cfDNA testing is currently more advanced in NIPT than in oncology, suggesting that oncological research could profit from experiences related to e.g. workflow standardization by NIPT. NIPT had already been globally adopted into clinical routines since 2015 [197], specifically for aneuploidy screening, and has since been implemented in more clinical practices and national healthcare systems worldwide, making it a highly standardized routine method [184].

In addition to the classical genetic aberrations, the assessment of additional features such as methylation and fragmentation patterns in ctDNA have gained attention in cancer research in recent years [146]. Similarly, cffDNA could hold valuable epigenetic information, with the potential to serve as disease biomarker. The analysis of DNA methylation is for example required for the diagnosis of imprinting disorders including the Angelman syndrome [198].

Apart from the use of cffDNA to study the fetal genome, recent studies suggest that cffDNA plays a functional role or can serve as a biomarker of several pregnancy-related comorbidities and complications including pregnancy loss, pre-eclampsia and pre-term labor [199]. Multiple studies could show that a low fetal fraction in cfDNA is associated with an increased risk of adverse pregnancy outcomes, especially hypertensive disorders [200]. In addition, excessive presence of feto-maternal microchimerism appears to correlate with the incidence of pre-eclampsia and fetal growth restrictions [12].

While cfDNA is more frequently used than circulating cells for the detection of genetic aberrations, CTC and circulating fetal cell analysis offer the unique possibility to study heterogeneity at a single cell level and gain functional insights into cell biology. In addition, genomic aberrations are detected with higher specificity due to the reduced background of maternal DNA. This is especially helpful for the analysis of maternally inherited mutations or for the analysis of the fetal methylation profiles.

Methods that are applied for the detection of circulating fetal cells are similar to the enrichment and detection methods of CTCs. Enrichment of circulating fetal cells is possible by density gradient centrifugation coupled with antibody cocktails for positive selection, depending on the cell type to be enriched. For instance, leukocytes can be detected via HLA [201, 202] or CD45 [202, 203], while premature nucleated red blood cells can be selected for using, among other possible markers, CD147 [204] or CD71 [205–207]. Other enrichment methods, such as MACS have also been used in combination with different targets and successfully enriched for fetal cells from maternal blood [66, 205, 208]. Importantly, very low false-positive rates have been described for healthy individuals or diseased controls with non-malignant diseases regarding circulating cells of epithelial origin [209]. For both, CTCs and circulating fetal cells, various markers are available for distinct subpopulations or tissues, respectively, and the choice of a marker or combination has to be carefully considered for each experiment [187]. Single circulating fetal cells can be manually picked and subjected to whole genome amplification for subsequent genotyping using custom next generation sequencing capture panels [4]. In principle, all methods developed for in-depth analysis of CTCs could be transferred to enriched circulating fetal cells, including single cell transcriptome or methylome analysis.

Next to CTCs and ctDNA, extracellular vesicles are currently investigated as additional analytes for liquid biopsies. Extracellular vesicle cargo is rich in protein, DNA, RNA and other molecules that can serve as biomarkers. Additionally, analysis of extracellular vesicles sheds light on the biology of cancer progression and metastasis, as multiple studies showed that actively secreted extracellular vesicles can prime tumor stroma or the premetastatic niche [210]. Similarly, extracellular vesicles have gained attention in feto-maternal crosstalk, as they are vertically transferred between the mother and the fetus. Extracellular vesicles can be found in higher quantities than circulating fetal cells and therefore hold the potential to serve as biomarkers for diseases that are associated with distorted placental metabolism or perfusion [211, 212].

While circulating fetal cell and cffDNA levels rapidly decrease after birth, a minority of fetal cells persist in the maternal organism even years after pregnancy [211]. The role of these cells and consequences for the maternal health are under debate, but multiple studies suggest a role in cancer [213] **(**Table 3**)**. A correlation of the presence of FMC with the incidence of cancer has been shown in several cancer entities. However, protective function as well as reduced cancer risks have been observed in different studies [151, 177, 214–220]. Therefore, the effects seem to be dependent on the tissue of origin and as most studies only analyze correlation of cancer incidence and the presence of FMC (often only based on Y chromosome sequence) more research is required to identify the functional reasons for the modulated cancer risk. Translating the knowledge from liquid biopsy as applied in oncology to the feto-maternal medicine field (and vice versa) could therefore support elucidating the functional role of circulating fetal cells and the biology of FMC.

**Table 3:** Modulation of Cancer Risk by feto-maternal microchimerism: Summary of observational studies on feto-maternal microchimerism (FMC) and cancer incidence, including the tumor entity, study cohort, number of patient samples analyzed, the method of FMC detection and the study outcome

Tumor Entity	Study subjects	*N* patients	FMC detection	Outcome	Reference
Brain Cancer	Case-control cohort	578	Y chromosome in blood	Reduced risk in FMC positive women	[214]
	Cancer patients	205	Y chromosome in tumor tissue	Unclear/ambiguous	[215]
Breast Cancer	Case-control cohort	38	Y chromosome in tumor tissue	Reduced risk in FMC positive women	[151]
	Case-control cohort	361	Y chromosome in blood	Reduced risk in FMC positive women	[216]
Cervical Cancer	Case-control cohort	15	Y chromosome in tumor tissue	FMC more frequent in cancer patients	[177]
Colorectal Cancer	Case-control cohort	339	Y chromosome in blood	Increased risk in FMC positive women	[216]
Endometrial Cancer	Case-control cohort	581	Y chromosome in blood	Reduced risk in FMC positive women	[217]
Ovarian Cancer	Case-control cohort	700	Y chromosome in blood	Reduced risk in FMC positive women	[218]
Skin Cancer	Cancer patients	24	Y chromosome in tumor tissue	Increased FMC in tumor	[219]
Thyroid Cancer	Cancer patients	63	Y chromosome in tumor tissue	Protective effect when FMC detected in tissue	[220]

## Conclusion

The analysis of CTCs and ctDNA in the blood of cancer patients has received tremendous attention over the past decade. Despite the different research fields, strong similarities between cancer research and feto-maternal medicine exist with respect to rare circulating cells and cell-free DNA. Both fields are confronted with low analyte concentration and a high biological background noise requiring highly sensitive analysis techniques. As of now, in the cancer field liquid biopsies do not present an alternative in economic regards [221], but they offer possibilities for screening, for example in patients with colorectal cancer that refused colonoscopy [222]. Moreover, recent recommendations from the European Medical Society working group state that patients with advanced cancer could get a blood-based genomic analysis for genomic aberrations relevant to drug treatment if tissue biopsies are unavailable [223]. Several organizations around the world including the European Liquid Biopsy Society (www.elbs.eu) are now in contact with the regulatory stakeholders to discuss the road map for integrating liquid biopsy in the reimbursement of health insurance systems. Despite the economic disadvantage also faced for NIPT liquid biopsy presents useful as a second line testing after routine screening [224].

Experience from cancer research could be utilized to advance prenatal testing in feto-maternal medicine and vice versa. Highly sensitivity techniques and lessons learned from cancer research can help in cffDNA detection and analysis, while the experience gained from routine application of cffDNA in NIPT can be transferred to clinical application of ctDNA in cancer patients. In depth characterization of circulating fetal cell populations, for instance by single cell analysis, as conducted in the CTC field, can support to unravel the underlaying mechanisms. However, more systematic studies are required to assess and understand the functional role of feto-maternal microchimerism and cancer. Despite the application in NIPT, analysis of cffDNA offers the possibility to deepen our understanding of the processes contributing to spontaneous preterm birth as well as to identify novel biomarkers to predict pregnancy associated complications.

## Data Availability

Data sharing not applicable to this article as no datasets were generated or analyzed during the current study.
